# Evaluating the Performance of Five Leading Large Language Models in Generating Health Education Materials for Patients With Infertility: Comparative Analysis

**DOI:** 10.2196/97878

**Published:** 2026-10-27

**Authors:** Renjie Lu, Yuchen Wei, Siyuan Wu, Yaning Han, Shenyu Zhao, Lingmin Hu, Yang Chen

**Affiliations:** 1 Changzhou Third People's Hospital, Changzhou Medical Center, Nanjing Medical University, Changzhou, Jiangsu, China; 2 Department of Reproduction, Changzhou Maternal and Child Health Care Hospital, Changzhou Medical Center, Nanjing Medical University, No 16 Dingxiang Road, Zhonglou District, Changzhou, Jiangsu, China, 86 (0519) 88581188; 3 School of Public Health, Xuzhou Medical University, Xuzhou, Jiangsu, China; 4 Tianjin University of Traditional Chinese Medicine, Tianjin, China; 5 The Affiliated Changzhou Hospital of Xuzhou Medical University, Changzhou Third People’s Hospital, Changzhou, Jiangsu, China

**Keywords:** large language models, infertility, health education, readability, content quality

## Abstract

**Background:**

Patients with infertility often have inadequate reproductive health literacy (RHL), while existing health education materials may be difficult to read, overly complex, and insufficiently tailored to individual needs. In recent years, large language models (LLMs) have created new opportunities to improve health education through their ability to generate and interpret medical information. However, their applicability and content quality in infertility education remain insufficiently evaluated.

**Objective:**

This study systematically evaluated the readability and content quality of LLM-generated health education materials for patients with infertility, identified model strengths and limitations, and provided evidence to support their standardized use in reproductive health.

**Methods:**

This study selected DeepSeek-R1, ChatGPT (GPT-5.2 Plus), Kimi 2.5, Qwen 3, and Gemini 3 as the study subjects. Based on the theoretical framework of RHL, this study developed a standardized question list comprising 6 dimensions and 93 items, and used unified prompts to generate health education texts in both Chinese and English. In this study, the Datayze Readability Analyzer was used to assess readability via the Flesch Reading Ease, Gunning Fog Index, and Flesch-Kincaid Grade Level (FKGL). Meanwhile, the content quality was evaluated in a double-blinded manner by reproductive medicine experts across 3 dimensions: accuracy, comprehensiveness, and humanistic care. Repeated-measures analysis of variance was used to compare performance across the LLMs, and Pearson correlation analysis was conducted to examine the associations between readability and content quality. A radar chart was used to evaluate the overall performance of each model.

**Results:**

The 5 LLMs differed significantly in both text readability and content quality. In terms of readability, Gemini 3 and Qwen 3 performed best, with significantly higher FRE scores than the other 3 models. DeepSeek-R1 and Kimi 2.5 performed least favorably, with FKGL scores corresponding to a ninth-grade reading level. ChatGPT (GPT-5.2 Plus) showed intermediate performance. In terms of content quality, Kimi 2.5 performed best across accuracy, comprehensiveness, and humanistic care, followed by DeepSeek-R1. ChatGPT (GPT-5.2 Plus) performed least favorably. Analysis revealed significant negative correlations between readability and content comprehensiveness for texts generated by Kimi 2.5, indicating a trade-off between these 2 attributes. The composite radar chart analysis showed that Kimi 2.5 achieved the highest score (11,594.33), whereas ChatGPT (GPT-5.2 Plus) had the lowest score (5555.83).

**Conclusions:**

Different LLMs showed significant differences in performance in generating health education texts for patients with infertility. In some models, readability was negatively correlated with content comprehensiveness. Under the conditions of this study, Kimi 2.5 demonstrated strong overall performance. However, no model achieved the best performance across all evaluation dimensions. Future applications of LLMs in reproductive health education will require domain-specific optimization, context-specific adaptation, and systematic quality control to support more accurate, comprehensive, and patient-centered communication.

## Introduction

In recent years, many countries around the world have been confronted with the dual challenge of persistently declining fertility rates and a rising prevalence of infertility [1]. Globally, approximately 17.5% (equivalent to approximately 1 in 6 people) of adults are affected by infertility [2]. From 2007 to 2020, the prevalence of infertility in China increased from 12% to 18% [3]. Despite the relatively high prevalence of infertility in China, the uptake of treatment remains comparatively low [4]. Possible explanations include not only objective barriers, such as limited access to medical resources, financial burden, and time costs, but also a widespread lack of reproductive health knowledge among people with infertility, which may be an important factor contributing to their low levels of health care seeking and treatment engagement.

Reproductive health literacy (RHL) is particularly important in the diagnosis and treatment of infertility [5]. Previous studies have proposed a theoretical framework for RHL based on the theoretical framework of health literacy [6]. RHL refers to the ability of individuals to access, understand, and apply information to make informed decisions about their reproductive health [7]. However, RHL has not received sufficient attention. In the course of seeking care, patients with infertility are often required to repeatedly interpret examination results and compare multiple treatment options [8,9]. Moreover, many women who are actively trying to conceive hold misconceptions about fertility and reproductive health [10]. However, insufficient RHL may result in misunderstanding of medical information, unrealistic treatment expectations, increased decisional conflict, and reduced efficiency of patient-provider communication, thereby adversely affecting the care experience and health outcomes [11-13]. Therefore, interventions to improve RHL among people of reproductive age, particularly those with infertility, are urgently needed.

The average reading ability of adults in the United States is approximately at the eighth-grade to ninth-grade level, whereas patient education materials are generally recommended to be written at the fifth to sixth-grade level [14]. In China, the Three-Year Action Plan for Improving Public Health Literacy (2024-2027) does not define specific grade-level readability criteria, but explicitly states that health information should be understandable to the public and aligned with the overarching goal of improving health literacy at the population level [15]. However, in reality, even professionally developed patient education materials often remain difficult to read, with reading demands that exceed the comprehension capacity of the general population [16-18]. The field of infertility encompasses a large volume of highly specialized information concerning reproductive physiological mechanisms, assisted reproductive technologies (ARTs), and long-term treatment decision-making [19-21]. Therefore, improving the readability and content quality of health education materials for patients with infertility is of considerable importance.

Generative AI, particularly large language models (LLMs), has gradually become an important channel for the public to access health information. Enabled by natural language interaction, these tools can respond to users’ health-related inquiries in real time, thereby effectively overcoming temporal and geographical barriers and providing patients with convenient access to informational support [22]. Moreover, through fine-tuning with human feedback, the natural language processing capabilities of LLMs have been markedly enhanced, enabling them to generate contextually appropriate and readily understandable responses to free-text prompts [23]. An increasing number of patients prefer to first obtain basic information through anonymized intelligent tools such as LLMs [24]. Information pertaining to infertility diagnosis and treatment is inherently highly sensitive and associated with substantial privacy concerns [25]. In the field of reproductive health, couples receiving infertility care tend to seek information through the internet and other intelligent information channels to address their informational needs [26]. Therefore, LLMs are expected to become an important tool for improving RHL among patients with infertility.

Although LLMs show considerable potential for simplifying specialized content and enhancing text readability, there are still concerns regarding their accuracy and comprehensiveness when handling complex medical issues [27]. Moreover, the content generated by different LLMs may differ substantially in terms of readability, accuracy, and medical consistency [28,29]. Previous studies have likewise demonstrated substantial differences in the readability and comprehensibility of health information generated by models such as ChatGPT (OpenAI), Gemini (Google DeepMind), and DeepSeek-R1 (Hangzhou DeepSeek Artificial Intelligence Co, Ltd) [30,31]. For patients with infertility, health education information must not only be easy to understand but also medically accurate and comprehensive. Therefore, systematically evaluating the quality of infertility-related health education materials generated by different LLMs is essential for determining their potential value in supporting patients’ reproductive health information needs. Guided by the RHL theoretical framework, this study developed a standardized question set based on clinical guidelines, frequently asked questions from patients, and the existing literature. Using this question set, we systematically compared the readability and content quality of infertility-related health education materials generated by 5 widely used LLMs: DeepSeek-R1, Kimi 2.5 (Moonshot AI), ChatGPT (GPT-5.2 Plus), Gemini 3, and Qwen 3 (Alibaba Cloud). Accordingly, this study proposed the following hypotheses: (1) the readability and overall content quality of infertility-related health education materials generated by the 5 LLMs may differ significantly; (2) LLM-generated reproductive health information may exhibit a trade-off between readability and content quality; and (3) differences in model architecture and optimization priorities may lead to distinct performance profiles across LLMs.

## Methods

### Study Subjects

This study selected 5 LLMs for evaluation: DeepSeek-R1, ChatGPT (GPT-5.2 Plus), Kimi 2.5, Qwen 3, and Gemini 3. Model selection was based on public accessibility, widespread use, and representativeness across different model types. In addition, the LLMs were selected to include both Chinese-developed models and internationally leading models, thereby enabling comparison of the readability and content quality of infertility-related health education texts generated by different LLMs. All text generation by the 5 LLMs was completed between February 7, 2026, and February 10, 2026. From February 12, 2026, to February 27, 2026, the Chinese and English texts generated by the LLMs underwent a double-blind quality evaluation conducted by reproductive medicine experts. Text readability was quantitatively assessed using standardized formulas. Content quality was evaluated in a double-blind review by reproductive medicine experts across 3 dimensions: accuracy, comprehensiveness, and humanistic care. The study design and research framework are presented in Figure 1.

**Figure 1.**
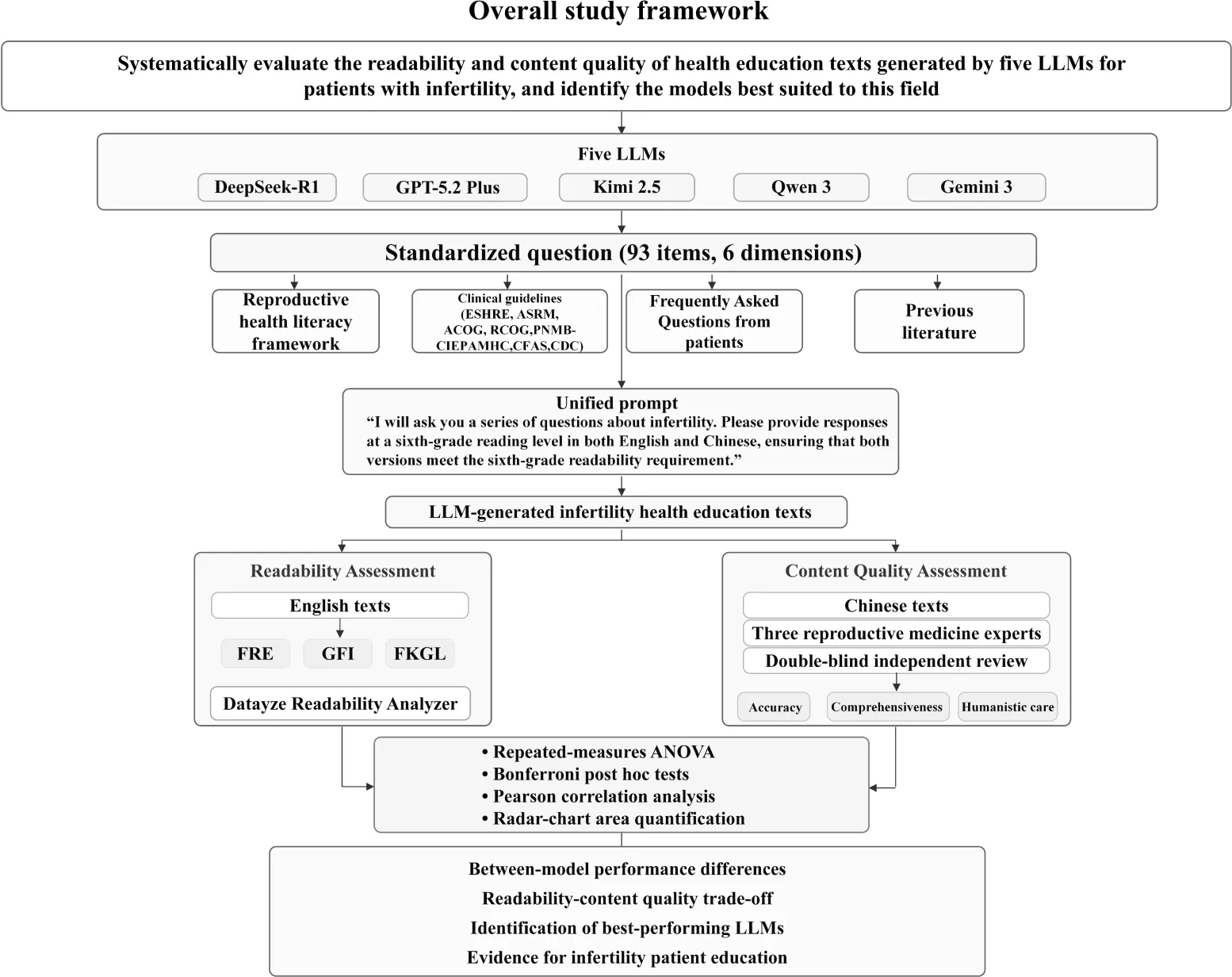
Study design and research framework. ACOG: American College of Obstetricians and Gynecologists; ANOVA: analysis of variance; ASRM: American Society for Reproductive Medicine; CDC: Centers for Disease Control and Prevention; CFAS: Canadian Fertility and Andrology Society; ESHRE: European Society of Human Reproduction and Embryology; FKGL: Flesch-Kincaid Grade Level; FRE: Flesch Reading Ease; GFI: Gunning Fog Index; LLM: large language model; PNMB-CIEPAMHC: Perinatal and Nutritional Metabolism Branch of the China International Exchange and Promotive Association for Medical and Health Care; RCOG: Royal College of Obstetricians and Gynaecologists.

### Ethical Considerations

This study was approved by the Ethics Committee of Changzhou Third People’s Hospital (approval number: 02A-A2025024).

### Development of the Question Set

Drawing on a theoretical framework, clinical guidelines, frequently asked questions from patients, and the existing literature, this study ultimately developed a standardized assessment question set comprising 6 dimensions and 93 items. Its development followed the principles of systematic categorization, evidence-based content, and evaluability. The study first established 6 dimensions based on a thematic framework of infertility-related health information needs among people of reproductive age: Physiological Risks and Disease-Related Factors; Family and Social Consequences; Medical Interventions and Health Improvement (Examinations, Treatment, and Assisted Reproductive Technologies); Resource and Financial Constraints (Costs and Health Insurance Coverage); External Triggers (Online Information, Family Members, and Friends); and Coping Self-Efficacy and Psychological Adjustment [32]. In addition, based on the 4 core competencies of the RHL framework, each dimension was further categorized into 4 assessment levels: access, understanding, appraisal, and application. This hierarchical evaluation framework guided the systematic development of a standardized question list comprising 93 items [6]. The development of the question list was primarily informed by official patient education guidelines issued by authoritative reproductive medicine organizations, including the European Society of Human Reproduction and Embryology [33-37], the American Society for Reproductive Medicine [38-40], the American College of Obstetricians and Gynecologists [41], the Royal College of Obstetricians and Gynaecologists [42], the Society for Assisted Reproductive Technology [43], the Perinatal and Nutritional Metabolism Branch of the China International Exchange and Promotive Association for Medical and Health Care [44], the Canadian Fertility and Andrology Society [45], and the Division of Reproductive Health of the Centers for Disease Control and Prevention [46].

### Generation of LLM Texts and Data Collection

Given the iterative evolution of LLM algorithms and the dynamic updating of their underlying knowledge bases, this study strictly narrowed the evaluation time window to ensure data homogeneity and comparability. All models were queried using a unified standardized prompt: “I will ask you a series of questions about infertility. Please provide responses at a sixth-grade reading level in both English and Chinese, ensuring that both versions meet the sixth-grade readability requirement.” Each LLM was instructed to independently generate health education materials in Chinese and English using the same standardized prompt. The text in one language was not translated to produce the corresponding version in the other language. This strategy was intended to preserve the natural language generation characteristics of different LLMs across linguistic contexts. It also minimized potential information loss, terminological changes, and alterations in writing style introduced during translation. It should be noted that the readability formulas used by the Datayze Readability Analyzer were developed based on English-specific features, including sentence length, syllable count, and syntactic structure. Direct application of these formulas to Chinese texts may therefore introduce methodological bias [47,48]. Therefore, this study adopted a bilingual evaluation strategy. Specifically, the English-language texts generated by each LLM were analyzed using the Datayze Readability Analyzer to quantitatively assess readability and ensure the linguistic applicability of the readability metrics. The Chinese-language texts were used solely for subsequent content quality assessment by reproductive medicine experts. Prior to evaluation, all collected texts underwent standardized preprocessing to remove meaningless characters, formatting irregularities, and responses that deviated from the intended question prompts. Text generation was conducted on February 7, 2026, for ChatGPT (GPT-5.2 Plus), Gemini 3, Qwen 3, and DeepSeek-R1, and on February 10, 2026, for Kimi 2.5. All LLMs generated responses using their default settings.

To help readers understand the standardized question list and the specific format of the texts generated by the LLMs, Table S1 in Multimedia Appendix 1 presents representative examples of the standardized assessment questions developed for this study and the corresponding responses generated by the 5 LLMs. The complete question set is provided in Multimedia Appendix 2.

### Readability Assessment

The English-language texts independently generated by the 5 LLMs were analyzed using the Datayze Readability Analyzer to assess readability. The Flesch Reading Ease (FRE), Gunning Fog Index (GFI), and Flesch-Kincaid Grade Level (FKGL) are widely used metrics for assessing the readability of patient education materials [49]. A sixth-grade reading level was used as the readability benchmark for LLM-generated health education materials. This benchmark was based primarily on recommendations in the US health communication literature that patient education materials should be written at or below a sixth-grade reading level to facilitate comprehension among individuals with limited health literacy [50,51]. Although this benchmark has been widely used in studies of English-language health information readability, its cross-cultural applicability to Chinese-language health education requires further validation. Given the lack of a unified grade-based readability standard for health information in China, a sixth-grade reading level was used as the reference threshold for readability assessment in this study.

The FRE score is calculated based on the ratio of syllable counts to sentence length, with scores ranging from 0 to 100. Higher scores indicate greater readability [52]. The score ranges correspond to specific educational levels in the United States: scores of 90 to 100 indicate a fifth-grade reading level, 70 to 80 indicate a middle-school level, 50 to 60 indicate a high-school level, 30 to 50 indicate a college level, and scores below 30 indicate the difficulty level of professional or academic texts. A score of 60 is commonly regarded as the threshold for plain English. In this study, we evaluated the extent to which each generated text deviated from this threshold.

GFI is calculated as 0.4 times the sum of the average sentence length and the percentage of complex words, with complex words defined as those containing 3 or more syllables [53]. GFI scores correspond to grade levels in the US education system, with scores of 6 to 8 indicating a middle-school level, 9 to 12 indicating a high-school level, and 13 to 16 indicating a college level. A GFI score of 8 is commonly used as an ideal reference threshold for public health communication. Scores above 12 suggest substantially increased textual complexity and may present comprehension difficulties for the general public.

FKGL is mapped to grade levels in the US education system. Lower values indicate lower reading difficulty [47]. The target threshold for FKGL is set at 6.0. This benchmark corresponds to a sixth-grade reading level and aligns with the internationally recommended standard widely used for patient education materials.

### Quality Assessment

#### Review Panel

The content quality assessment team comprised 3 reproductive medicine specialists. Because the study focused on the medical accuracy, content comprehensiveness, and humanistic care of LLM-generated health education materials, clinical experts with expertise in reproductive medicine were selected as evaluators. Among the expert evaluators, 2 physicians had more than 10 years of clinical experience and held associate senior professional titles. The third physician held a doctoral degree in medicine, had more than 5 years of professional experience, and held an intermediate professional title. The inclusion of this younger physician was intended to better reflect the cognitive characteristics of younger patients and their discourse patterns during interactions with LLMs. In addition, a 2-member adjudication panel composed of reproductive medicine experts was established to provide a final resolution in cases of rating disagreements.

#### Standardized Training

Before the formal evaluation, all reviewers received standardized training to minimize individual subjective bias as much as possible. The training primarily covered the study background, evaluation objectives, and requirements for bias control; standardized the quantitative scoring criteria and decision thresholds; specified the procedures for double-blind review and score-sheet completion; and clarified the mechanism for resolving disagreements. After completing the training, a mock review exercise was conducted, and only those reviewers who met the predefined requirements were allowed to participate in the formal evaluation.

Content quality was evaluated across 3 dimensions: accuracy, comprehensiveness, and humanistic care. Accuracy was defined as the extent to which the generated texts were consistent with current clinical practice guidelines and evidence-based medicine. A high level of accuracy indicated the absence of professional factual errors or inappropriate diagnostic and therapeutic recommendations. Comprehensiveness was defined as the extent to which the generated texts adequately covered the core elements of the question. Comprehensiveness should encompass both medical information and practical health guidance. Humanistic care was defined as the extent to which the generated texts reflected respect for and understanding of the psychological state of patients with infertility. It was characterized by empathic expression, emotional support, and positive guidance.

#### Assessment Procedures

A double-blind, independent, multilevel review process was adopted in this study. Throughout the evaluation, the reviewers were blinded to the identity of the LLM responsible for generating each text. In addition, question numbers and reviewer-identifying information were concealed to minimize potential model-related bias and subjective preconceptions during the evaluation process. Two reviewers independently performed quantitative scoring and provided qualitative comments on the texts generated by each LLM. Items with substantial discrepancies in the first-round ratings were submitted to a third reviewer for reassessment. If disagreements persisted, additional experts were involved in the review until consensus was reached.

### Statistical Analysis

All statistical analyses were conducted using GraphPad Prism 10 and SPSS (version 27.0; IBM Corp). Tests of normality were performed before the formal data analysis. First, repeated-measures analysis of variance (RM-ANOVA) was performed to compare differences among texts generated by the 5 LLMs in terms of readability and content quality. When the sphericity assumption was not met, Greenhouse-Geisser or Huynh-Feldt corrections were applied as appropriate. When the overall effect was statistically significant, Bonferroni-adjusted post hoc comparisons were performed for pairwise testing. The purpose of using RM-ANOVA together with post hoc testing was to examine between-model differences across individual dimensions and to stratify the LLMs according to performance. Subsequently, Pearson correlation analysis was performed to examine the relationship between the readability and content quality of the generated texts, with the aim of identifying factors that may promote or hinder the effectiveness of patient education materials. To provide an integrated evaluation of model performance, all indicators were positively standardized to a 0 to 100 scale and presented in standardized radar charts. Radar charts generated in Origin 2021 (OriginLab Corp) were used for visualization and area quantification to identify the best-performing model. All statistical tests were 2-sided, with *P*<.05 considered statistically significant. For post hoc pairwise comparisons, adjusted *P* values were reported, and an adjusted *P*<.05 was used as the threshold for statistical significance.

## Results

### Readability

RM-ANOVA was conducted separately for the readability indices (FRE, GFI, and FKGL) across texts generated by the 5 LLMs. The Mauchly test of sphericity for both FRE and GFI indicated that the data did not meet the sphericity assumption (*P=*.009 *and P*<.001). Therefore, the Greenhouse-Geisser and Huynh-Feldt corrections were applied. The results of the within-subjects effects tests were *F* values of 91.102 and 81.073 (both *P*<.001). Mauchly test of sphericity for FKGL indicated that the sphericity assumption was met (*P*=.07), and the results of the within-subjects effects test were *F* value of 78.017 (*P*<.001).

Mean FRE scores were ranked from highest to lowest as Gemini 3, Qwen 3, ChatGPT (GPT-5.2 Plus), Kimi 2.5, and DeepSeek-R1 (Table 1; Table S2 in Multimedia Appendix 1). Bonferroni-adjusted post hoc comparisons indicated no statistically significant difference between Gemini 3 and Qwen 3 (*P*>.99). However, both models had significantly higher FRE scores than ChatGPT (GPT-5.2 Plus), Kimi 2.5, and DeepSeek-R1 (all *P*<.001).

**Table 1.** Readability of texts generated by the 5 large language models.

Model	FRE^a^, mean (SD)	GFI^b^, mean (SD)	FKGL^c^, mean (SD)
DeepSeek-R1	50.521 (14.625)	12.512 (2.820)	9.381 (2.417)
GPT-5.2 Plus	62.754 (19.138)	8.166 (3.549)	5.790 (2.617)
Kimi 2.5	51.613 (16.066)	11.668 (2.917)	8.807 (2.404)
Qwen 3	73.810 (12.565)	7.816 (2.626)	5.792 (2.337)
Gemini 3	75.451 (11.415)	8.370 (2.287)	5.725 (2.037)

a FRE: Flesch Reading Ease.

b GFI: Gunning Fog Index.

c FKGL: Flesch-Kincaid Grade Level.

Mean GFI scores were ranked from highest to lowest as DeepSeek-R1, Kimi 2.5, Gemini 3, ChatGPT (GPT-5.2 Plus), and Qwen 3 (Table 1; Table S2 in Multimedia Appendix 1). Bonferroni-adjusted post hoc comparisons indicated no statistically significant difference between DeepSeek-R1 and Kimi 2.5 (*P*=.08), whereas both models had significantly higher GFI scores than Gemini 3, ChatGPT (GPT-5.2 Plus), and Qwen 3 (all *P*<.001). Pairwise comparisons among Gemini 3, ChatGPT (GPT-5.2 Plus), and Qwen 3 revealed no statistically significant differences (*P*>.99, *P*=.65, and *P*>.99).

Mean FKGL scores were ranked from highest to lowest as DeepSeek-R1, Kimi 2.5, Qwen 3, ChatGPT (GPT-5.2 Plus), and Gemini 3 (Table 1; Table S2 in Multimedia Appendix 1). Bonferroni-adjusted post hoc comparisons indicated no statistically significant difference between DeepSeek-R1 and Kimi 2.5 (*P*=.468), whereas both models had significantly higher FKGL scores than Qwen 3, ChatGPT (GPT-5.2 Plus), and Gemini 3 (all *P*<.001). Pairwise comparisons among Qwen 3, ChatGPT (GPT-5.2 Plus), and Gemini 3 revealed no statistically significant differences (all *P*>.99).

To visually present the distributional characteristics and between-model differences in the readability of texts generated by the 5 LLMs, violin plots were used (Figures 2–4).

**Figure 2.**
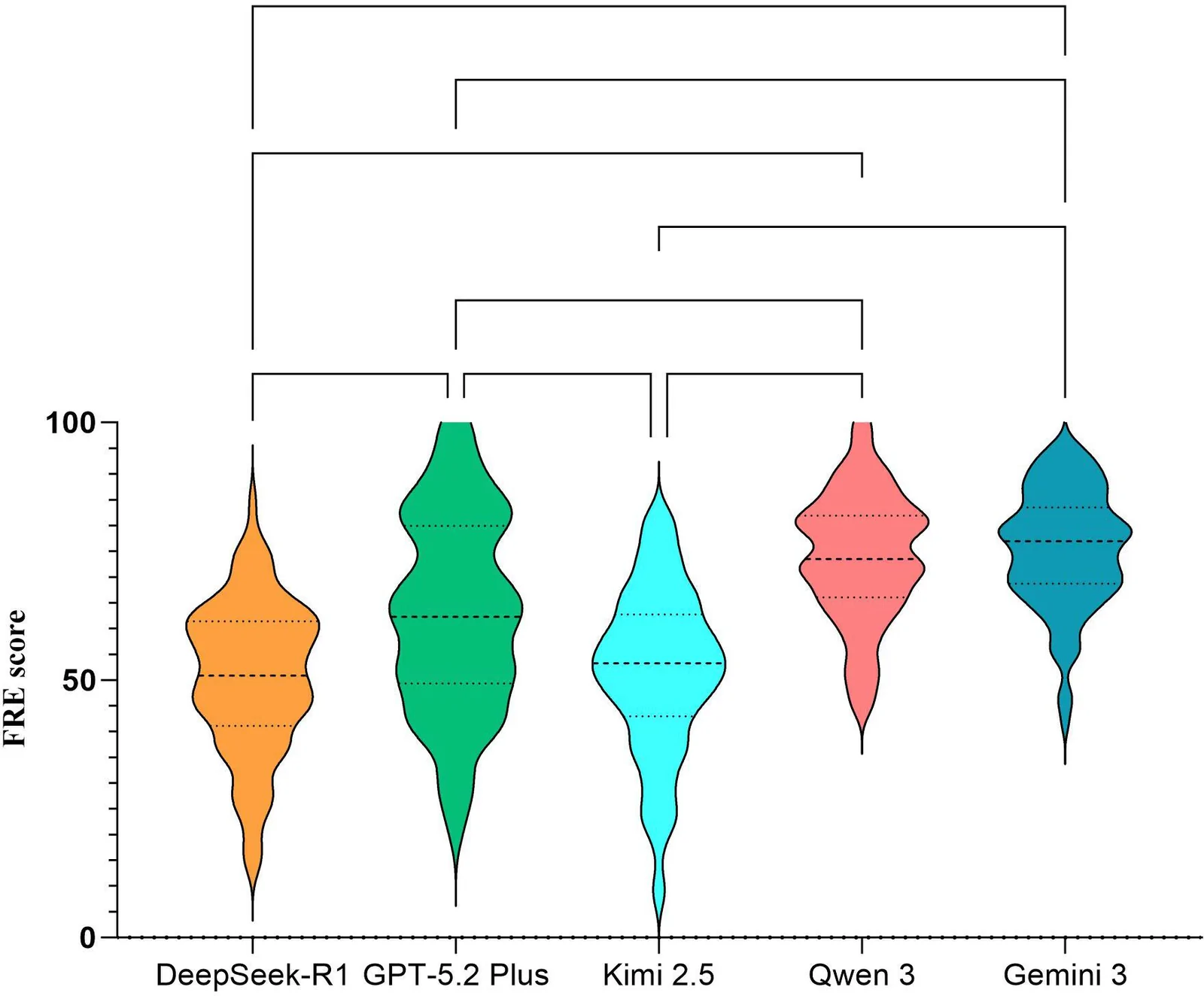
Readability comparison of texts generated by the 5 large language models displaying the Flesch Reading Ease (FRE) score. Horizontal lines above the violin plots indicate statistically significant differences between the corresponding models (*P*<.001).

**Figure 3.**
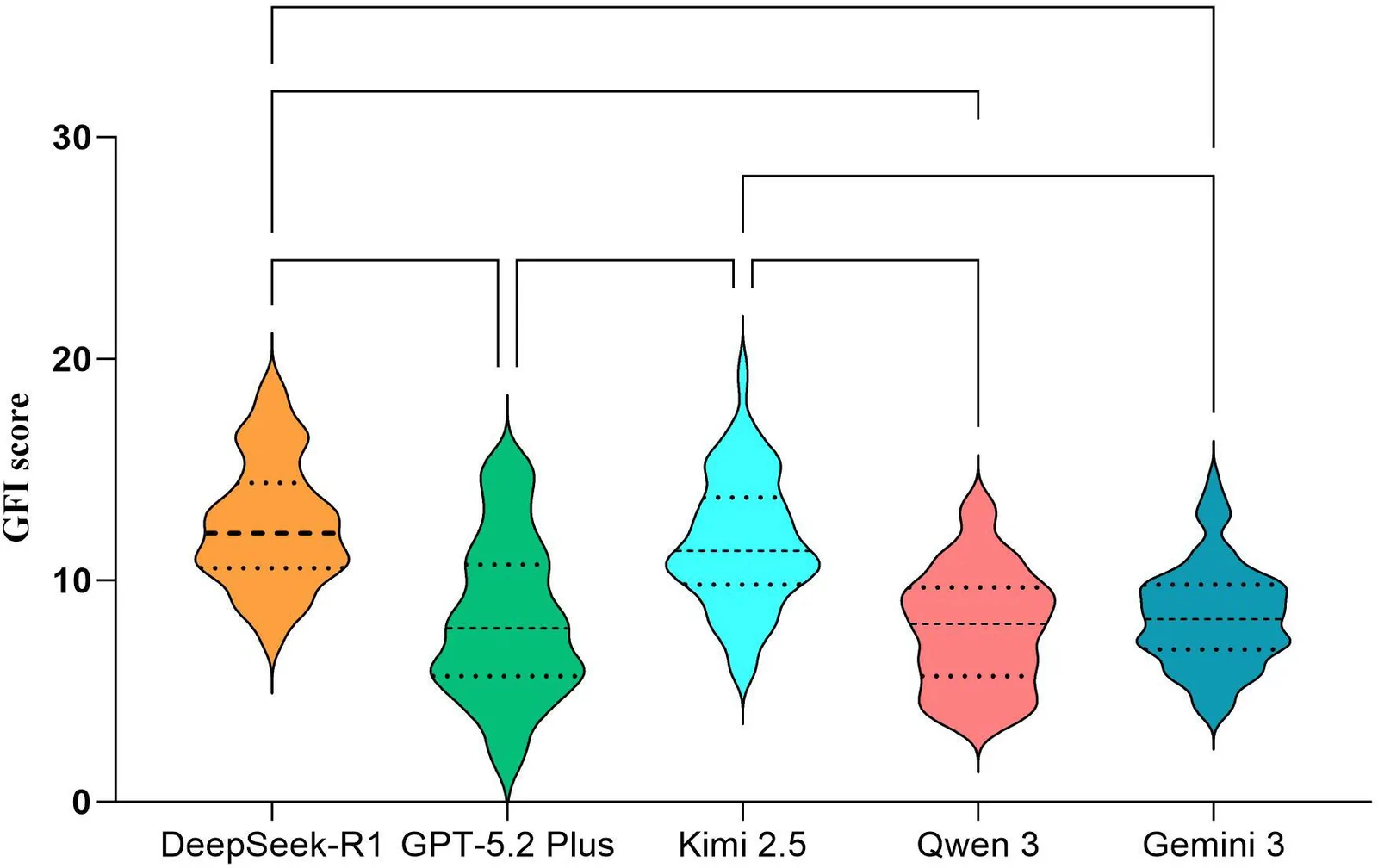
Readability comparison of texts generated by the 5 large language models displaying the Gunning Fog Index (GFI) score. Horizontal lines above the violin plots indicate statistically significant differences between the models (*P*<.001).

**Figure 4.**
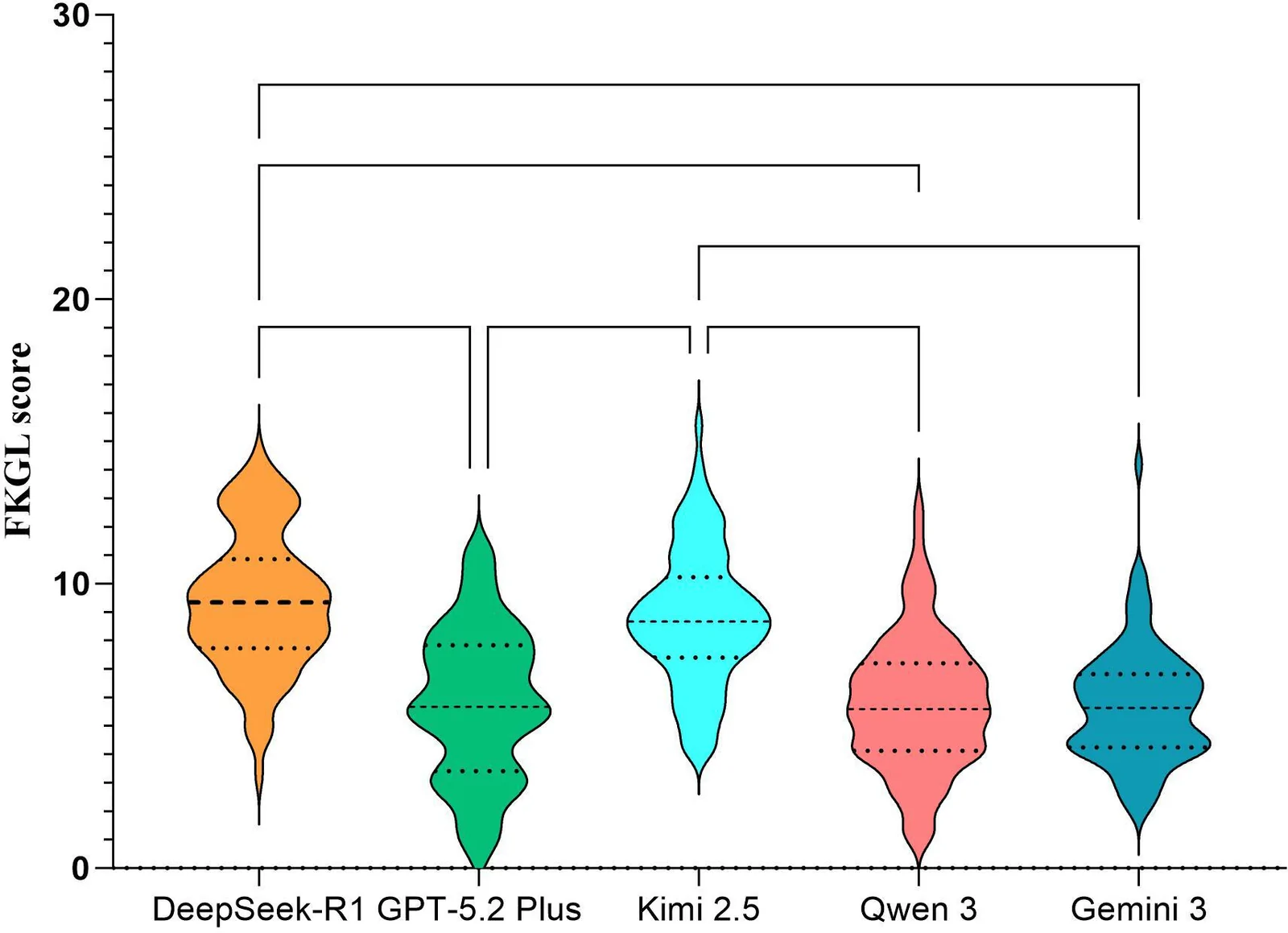
Readability comparison of texts generated by the 5 large language models displaying the Flesch-Kincaid Grade Level (FKGL) score. Horizontal lines above the violin plots indicate statistically significant differences between the corresponding models (*P*<.001).

### Content Quality Assessment

RM-ANOVA was conducted separately for the accuracy, comprehensiveness, and humanistic care in texts generated by the 5 LLMs. In Mauchly test of sphericity for accuracy, comprehensiveness, and humanistic care, all results indicated that the data failed to satisfy the sphericity assumption (*P*<.001, *P*=.002, and *P*<.001). Greenhouse-Geisser and Huynh-Feldt corrections were used, respectively, and the within-subjects effects test yielded *F* values of 41.191, 108.654, and 153.187 (all *P*<.001).

Mean accuracy scores were highest for Kimi 2.5, followed by DeepSeek-R1, Gemini 3, Qwen 3, and ChatGPT (GPT-5.2 Plus) (Tables 2 and 3). Bonferroni-adjusted post hoc comparisons indicated statistically significant differences between Kimi 2.5 and DeepSeek-R1 (*P*=.007), whereas both models achieved significantly higher accuracy scores than Gemini 3 (*P*<.001 and *P*=.002), Qwen 3, and ChatGPT (GPT-5.2 Plus) (both *P*<.001). Gemini 3 had significantly higher accuracy scores than ChatGPT (GPT-5.2 Plus) (*P*<.001), whereas the difference between Gemini 3 and Qwen 3 was not statistically significant (*P*=.10). Likewise, no statistically significant difference was observed between ChatGPT (GPT-5.2 Plus) and Qwen 3 (*P*=.63).

**Table 2.** Content quality of texts generated by the 5 large language models.

Model	Accuracy, mean (SD)	Comprehensiveness, mean (SD)	Humanistic care, mean (SD)
DeepSeek-R1	4.247 (0.481)	4.624 (0.509)	4.462 (0.523)
GPT-5.2 Plus	3.699 (0.484)	3.495 (0.544)	3.226 (0.513)
Kimi 2.5	4.452 (0.542)	4.699 (0.461)	4.817 (0.389)
Qwen 3	3.817 (0.607)	3.602 (0.610)	4.548 (0.562)
Gemini 3	4.011 (0.313)	3.914 (0.545)	4.118 (0.486)

**Table 3.** Post hoc pairwise comparisons of content quality among texts generated by the 5 large language models.

Comparison	Accuracy			Comprehensiveness			Humanistic care		
	Mean difference (95% CI)	SE	*P* value	Mean difference (95% CI)	SE	*P* value	Mean difference (95% CI)	SE	*P* value
DS^a^ vs CG^b^	0.548 (0.359 to 0.738)	0.066	<.001	1.129 (0.926 to 1.332)	0.070	<.001	1.237 (1.011 to 1.463)	0.079	<.001
DS vs KM^c^	−0.204 (−0.372 to −0.037)	0.058	.007	−0.075 (−0.241 to 0.091)	0.058	>.99	−0.355 (−0.523 to −0.187)	0.059	<.001
DS vs Q^d^	0.430 (0.208 to 0.652)	0.077	<.001	1.022 (0.761 to 1.282)	0.090	<.001	−0.086 (−0.295 to 0.123)	0.073	>.99
DS vs G^e^	0.237 (0.064 to 0.409)	0.060	.002	0.710 (0.501 to 0.919)	0.073	<.001	0.344 (0.140 to 0.548)	0.071	<.001
CG vs KM	−0.753 (−0.971 to −0.534)	0.076	<.001	−1.204 (−1.408 to −1.000)	0.071	<.001	−1.591 (−1.769 to −1.414)	0.062	<.001
CG vs Q	−0.118 (−0.299 to 0.062)	0.063	.63	−0.108 (−0.338 to 0.123)	0.080	>.99	−1.323 (−1.564 to −1.081)	0.084	<.001
CG vs G	−0.312 (−0.476 to −0.147)	0.057	<.001	−0.419 (−0.632 to −0.207)	0.074	<.001	−0.892 (−1.096 to −0.689)	0.071	<.001
KM vs Q	0.634 (0.424 to 0.844)	0.073	<.001	1.097 (0.856 to 1.338)	0.084	<.001	0.269 (0.082 to 0.456)	0.065	<.001
KM vs G	0.441 (0.237 to 0.645)	0.071	<.001	0.785 (0.566 to 1.004)	0.076	<.001	0.699 (0.530 to 0.868)	0.059	<.001
Q vs G	−0.194 (−0.406 to 0.019)	0.074	.10	−0.312 (−0.557 to −0.067)	0.085	.004	0.430 (0.204 to 0.656)	0.079	<.001

a DS: DeepSeek-R1.

b CG: GPT-5.2 Plus.

c KM: Kimi 2.5.

d Q: Qwen 3.

e G: Gemini 3.

Mean comprehensiveness scores were highest for Kimi 2.5, followed by DeepSeek-R1, Gemini 3, Qwen 3, and ChatGPT (GPT-5.2 Plus) (Tables 2 and 3). Bonferroni-adjusted post hoc comparisons showed no statistically significant difference between Kimi 2.5 and DeepSeek-R1 (*P*>.99), whereas both models had significantly higher comprehensiveness scores than Gemini 3, Qwen 3, and ChatGPT (GPT-5.2 Plus) (all *P*<.001). No statistically significant difference was observed between Qwen 3 and ChatGPT (GPT-5.2 Plus) (*P*>.99), and both models had significantly lower comprehensiveness scores than Gemini 3 (*P*=.004 and *P*<.001).

Mean humanistic care scores were highest for Kimi 2.5, followed by Qwen 3, DeepSeek-R1, Gemini 3, and ChatGPT (GPT-5.2 Plus), as shown in Tables 2 and 3. Bonferroni-adjusted post hoc comparisons indicated that Kimi 2.5 scored significantly higher than each of the other 4 models (all *P*<.001). No statistically significant difference was observed between DeepSeek-R1 and Qwen 3 (*P*>.99), whereas both models scored significantly higher than Gemini 3 and ChatGPT (GPT-5.2 Plus) (all *P*<.001). Gemini 3 scored significantly higher than ChatGPT (GPT-5.2 Plus) (*P*<.001).

### Correlation Analysis Between Readability and Content Quality

The correlation analysis indicated that a consistent positive association between readability and content quality was not observed across the texts generated by the models. For DeepSeek-R1, ChatGPT (GPT-5.2 Plus), and Qwen 3, no significant correlations were found between readability and content quality. For Kimi 2.5, FRE was significantly negatively correlated with comprehensiveness (*r*=–0.220; *P*=.03), whereas GFI was significantly positively correlated with comprehensiveness (*r*=0.272; *P*=.008). For Gemini 3, FKGL was likewise significantly negatively correlated with comprehensiveness (*r*=–0.229; *P*=.03). These findings suggested that a trade-off might exist between readability and content quality in the texts generated by Kimi 2.5. For Gemini 3, easier reading was associated with higher comprehensiveness. (Table 4).

**Table 4.** Correlation between readability and content quality of texts generated by the 5 large language models.

Model	FRE^a^		GFI^b^		FKGL^c^	
	*r^d^*	*P* value	*r*	*P* value	*r*	*P* value
DeepSeek-R1						
Accuracy	−0.115	.27	0.063	.55	0.135	.20
Comprehensiveness	0.030	.77	−0.009	.93	−0.068	.52
Humanistic care	0.078	.46	−0.036	.73	−0.119	.26
GPT-5.2 Plus						
Accuracy	−0.117	.26	0.167	.11	0.139	.18
Comprehensiveness	−0.029	.79	0.080	.45	0.045	.67
Humanistic care	0.070	.51	0.013	.90	−0.026	.80
Kimi 2.5						
Accuracy	−0.125	.23	0.096	.36	0.107	.31
Comprehensiveness	−0.220	.03	0.272	.008	0.201	.05
Humanistic care	0.121	.25	−0.117	.26	−0.126	.23
Qwen 3						
Accuracy	−0.055	.60	0.155	.14	0.155	.14
Comprehensiveness	−0.059	.57	0.181	.08	0.180	.08
Humanistic care	−0.065	.54	0.027	.80	0.055	.60
Gemini 3						
Accuracy	0.058	.58	−0.083	.43	−0.004	.97
Comprehensiveness	0.181	.08	−0.174	.10	−0.229	.03
Humanistic care	0.119	.26	−0.185	.08	−0.125	.23

a FRE: Flesch Reading Ease.

b GFI: Gunning Fog Index.

c FKGL: Flesch-Kincaid Grade Level.

d *r*: Pearson correlation coefficient.

### Integrated Performance Analysis

Radar charts were constructed based on positively standardized scores on a 0 to 100 scale, and the polygon area of each radar chart was used to quantify the overall performance of the 5 LLMs (Figure 5). Texts generated by Kimi 2.5 and DeepSeek-R1 performed strongly in terms of accuracy, comprehensiveness, and humanistic care but showed poorer performance on readability metrics. By contrast, Gemini 3 performed best in readability but received comparatively lower scores for content quality. Qwen 3 also demonstrated favorable overall readability, although it showed no clear advantage in content quality. ChatGPT (GPT-5.2 Plus) performed comparatively poorly across multiple evaluation dimensions and therefore showed limited overall performance.

A larger radar chart area indicates superior overall model performance [54-56]. Quantitative analysis of the radar chart areas showed that Kimi 2.5 achieved the highest overall score (11,594.33), followed by DeepSeek-R1 (9926.07), Gemini 3 (8487.88), and Qwen 3 (7885.54). ChatGPT (GPT-5.2 Plus) had the lowest overall score (5555.83), as shown in Table 5.

**Figure 5.**
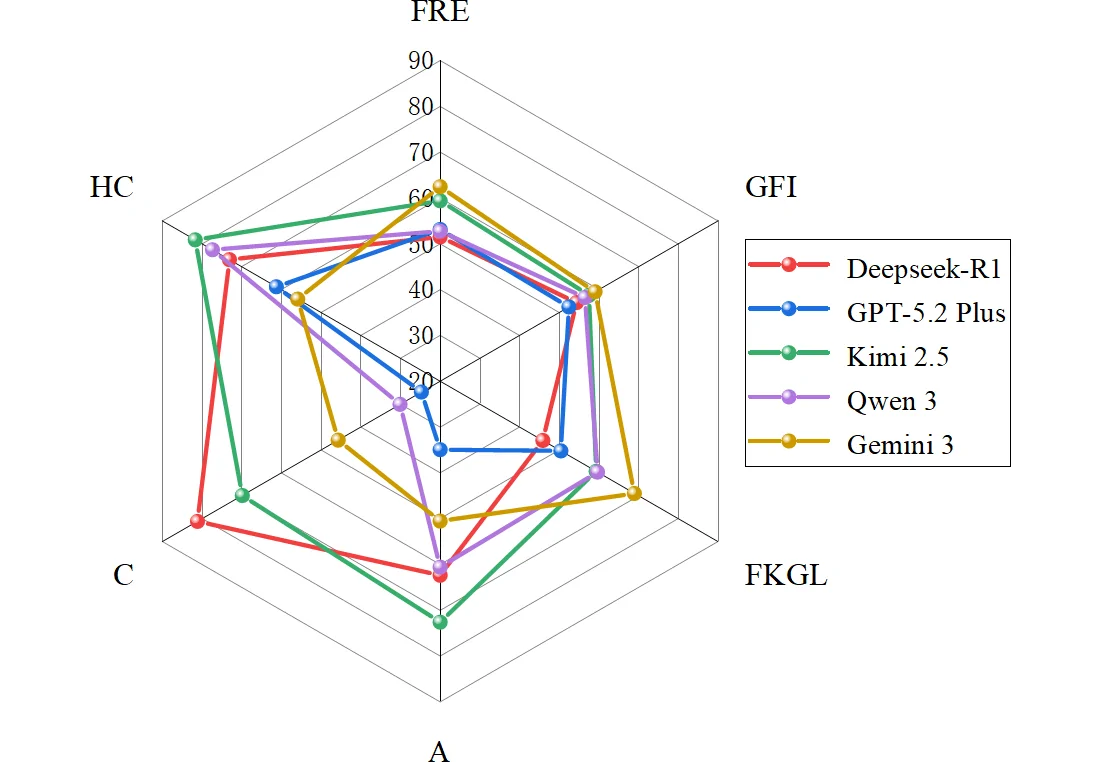
Comparison of integrated performance across texts generated by the 5 large language models. A: accuracy; C: comprehensiveness; HC: humanistic care; FKGL: Flesch-Kincaid Grade Level; FRE: Flesch Reading Ease; GFI: Gunning Fog Index.

**Table 5.** Radar chart area and ranking for the integrated performance of texts generated by the 5 large language models.

Model	Radar chart area	Overall ranking
Kimi 2.5	11,594.33	1
DeepSeek-R1	9926.07	2
Gemini 3	8487.88	3
Qwen 3	7885.54	4
GPT-5.2 Plus	5555.83	5

## Discussion

### Principal Findings

Using a progressive analytical framework that incorporated between-model differences, interdimensional associations, and multidimensional integration, this study systematically evaluated the readability and content quality of texts generated by 5 LLMs, thereby providing an evidence base to guide the scientific selection of LLMs best suited to health education for patients with infertility.

This study assessed the readability of LLM-generated texts using FKGL, FRE, and GFI, and adopted a sixth-grade reading level as the reference threshold. This threshold was based on the American Medical Association’s recommendations regarding the readability of patient education materials [17]. China currently lacks official readability standards based on educational grade levels issued by authorities such as the National Health Commission. However, this does not mean that China lacks a system for evaluating the quality of health information. On the contrary, the development of China’s health information evaluation system has followed a different path from that of Europe and North America. It places greater emphasis on the overall health literacy of the population and the quality of health education materials [57]. At the instrument level, Chinese text readability tools, such as AlphaReadabilityChinese, have been developed. However, grading standards corresponding to the public’s educational reading levels have not yet been established [58]. Although the Chinese version of the Patient Education Materials Assessment Tool for Printable Materials has been translated and validated, it assesses the understandability and actionability of materials rather than their reading level [59,60]. These findings indicate that China currently lacks a health information readability grading system that can be directly aligned with the public’s reading ability. Therefore, the use of internationally recognized standards as reference thresholds in this study represented a pragmatic interim approach. This limitation may affect the precise assessment and context-specific interpretation of the readability of Chinese-language infertility health education materials generated by different LLMs. It also highlights the need to develop readability standards tailored to the Chinese linguistic context to enable more accurate evaluation of the effectiveness of LLMs in patient education in China.

The findings suggest that the texts generated by DeepSeek-R1 and Kimi 2.5 both showed significant disadvantages across the 3 readability metrics, with FKGL scores corresponding to a ninth-grade reading level. Previous studies have similarly shown that DeepSeek-R1 performs relatively poorly in terms of readability, generating patient education materials that are significantly more complex than those produced by Gemini and ChatGPT [61,62]. Other studies have similarly reported that the FKGL score of the texts generated by Kimi can even reach the 13th- to 14th-grade level [63]. The poor readability performance of the texts generated by DeepSeek-R1 and Kimi 2.5 may reflect an architectural tendency to prioritize information density. The use of more complex terminology and longer sentence structures may limit their utility for patients with infertility who have lower levels of health literacy. In addition, the texts generated by ChatGPT (GPT-5.2 Plus) demonstrated intermediate readability performance. By contrast, the texts generated by Gemini 3 and Qwen 3 showed clear readability advantages. The texts generated by both models achieved significantly higher FRE scores than the other 3 LLMs, had FKGL values approaching the prespecified sixth-grade target, and exhibited relatively low GFI scores. Previous studies have likewise demonstrated a clear advantage of Gemini 3 in text simplification [28,64].

With respect to accuracy and comprehensiveness, the texts generated by DeepSeek-R1 and Kimi 2.5 outperformed Qwen 3 and Gemini 3. DeepSeek-R1’s strong performance across these 2 dimensions may be closely attributable to its deep-reasoning architecture (DeepSeek-R1-Zero), which supports multistep logical verification through chain-of-thought reasoning and may therefore facilitate more complete outputs for complex medical information [61]. With respect to humanistic care, the texts generated by DeepSeek-R1 and Qwen 3 performed slightly better than Gemini 3, but remained less effective than Kimi 2.5. The moderate performance of DeepSeek-R1-generated texts in the humanistic care dimension suggests that an excessive architectural emphasis on logical rigor in LLMs may come at the expense of the natural expression of emotional resonance.

Compared with DeepSeek-R1, Qwen 3, and Gemini 3, the texts generated by Kimi 2.5 demonstrated superior content quality. Its underlying optimization strategy may be more closely aligned with the demands of medical communication scenarios. Previous studies have shown that these technical approaches can improve the factual quality of outputs in knowledge-intensive tasks and enhance the empathic performance of dialogue systems, respectively [65-67]. Although the texts generated by Qwen 3 and Gemini 3 demonstrated slightly better readability than DeepSeek-R1 and Kimi 2.5, their content quality was comparatively lower. This suggests that differences in architectural design and optimization priorities across LLMs may underlie their distinct performance profiles.

The texts generated by ChatGPT (GPT-5.2 Plus) exhibited the poorest content quality among those generated. This may be attributable to insufficient training on non-English medical corpora, heterogeneity in clinical guidelines across countries, limited adaptation to the Chinese cultural context, and inadequate domain-specific alignment for specialized areas such as infertility and reproductive health. Similar performance limitations have likewise been reported in previous studies. For example, in the 2022 China National Medical Licensing Examination, ChatGPT scored only 51 points, falling below the passing threshold [68]. ChatGPT also failed to pass the Finnish written examination in plastic surgery [69]. ChatGPT also achieved only 65% accuracy on the official board examination of the Japan Radiological Society [70]. Therefore, the deployment of LLMs in cross-linguistic, cross-cultural, and specialty-specific medical settings requires dedicated optimization.

For Kimi 2.5 , higher FRE was associated with lower comprehensiveness and higher GFI with higher comprehensiveness, supporting a readability-comprehensiveness trade-off. These findings suggest a potential trade-off relationship between readability and content quality in LLM-generated texts, where an increase in one was accompanied by a decrease in the other. One evaluation study of radiology reports generated by ChatGPT (GPT-4) found that when the reports were simplified to a seventh-grade reading level, 20% of the reports contained inaccuracies with the potential to affect clinical decision-making [71]. Efforts to improve text readability often involve linguistic simplification, which may lead to the loss of key information and, consequently, compromise content quality [72]. Conversely, when LLMs attempt to provide more comprehensive and in-depth information, they may inevitably use more specialized terminology and more complex sentence structures, thereby diminishing text readability. Overall, these findings suggest that LLMs may not yet have fully achieved an optimal balance between linguistic simplification and the complete and accurate communication of specialized medical knowledge.

The radar chart analysis further corroborated the above findings. Quantification of radar chart area showed that Kimi 2.5 achieved the highest overall score. Although the texts generated by Kimi 2.5 were somewhat less favorable in terms of readability, the model’s substantial advantage in content quality suggests that it may currently be the preferred model for supporting health education for patients with infertility. The texts generated by DeepSeek-R1 exhibit a typical pattern of high content quality but low readability. The texts generated by Qwen 3 and ChatGPT (GPT-5.2 Plus) ranked lower in overall performance, in that order. The texts generated by Kimi 2.5 and DeepSeek-R1 may be suitable for patient education in infertility care after plain-language refinement by reproductive medicine clinicians. By contrast, texts generated by Gemini 3 and Qwen 3 would require more rigorous review, modification, and supplementation by reproductive medicine clinicians before being considered appropriate for patient education. Therefore, whenever LLMs are used in health education for patients with infertility, essential human oversight should be incorporated, including professional interpretation, content review, and corrective revision. Overall, different LLMs exhibit distinct performance profiles, and no single model currently achieves optimal performance in both readability and content quality. Therefore, when LLMs are used to provide health education for patients with infertility, review by reproductive medicine experts, content correction, and plain-language adaptation remain necessary to ensure the accuracy, understandability, and safety of the health information provided.

### Practical Implications

Balancing content quality with readability may represent an important direction for the continued optimization of LLMs. Nevertheless, given current technological constraints, the selection and deployment of LLMs should remain differentiated according to the risk level of the specific application scenario. In high-risk, highly specialized scenarios, such as the optimization of ART protocols, LLMs with stronger overall performance (eg, Kimi 2.5 and DeepSeek-R1) may be prioritized for generating health education texts. These generated texts may function as real-time supportive tools for reproductive medicine clinicians in the delivery of patient education. In lower-risk health communication scenarios, such as dietary guidance, LLMs with better readability (eg, Qwen 3 and Gemini 3) may be more suitable for generating patient education materials. However, this study suggests that texts generated by these LLMs should be subject to rigorous review and revision by reproductive medicine clinicians. At the organizational level, AI should be positioned as a tool for cognitive augmentation rather than a replacement, with a dual-governance framework of “AI-assisted generation and human-led verification” established to support its use [72]. Furthermore, before internationally mainstream LLMs (eg, ChatGPT) are deployed in Chinese clinical practice, they should undergo systematic validation of Chinese medical terminology, adaptation to Chinese linguistic conventions, and assessment of psychological sensitivity. Meanwhile, this study recommends that model developers optimize Chinese-language medical corpora for the field of infertility, dynamically update clinical guidelines, and enhance adaptability to cultural contexts.

### Theoretical Implications

This study developed a standardized evaluation framework and methodological system for the application of LLMs in reproductive health education, thereby addressing a gap in quantitative research on LLM performance in the specialty context of infertility. In addition, this study is the first to identify a trade-off between readability and content quality in LLM-generated reproductive health education texts, highlighting the inherent tension between the simplified communication of medical information and the high-quality transmission of specialized knowledge. These findings provide a theoretical basis for further optimization of LLM performance in the field of reproductive health. Grounded in the theoretical framework of RHL, this study integrated model performance evaluation with the real-world informational needs of patients with infertility and established a specialty-specific evaluation system, thereby providing a paradigmatic reference for future studies on the adaptability of LLMs across different medical specialties. In addition, this study demonstrated both the feasibility and heterogeneity of LLMs as tools for improving RHL, thereby offering theoretical support for the transformation of health literacy interventions from traditional offline approaches to intelligent digital modalities.

### Limitations

This study has several limitations. First, the FRE, GFI, and FKGL formulas used to assess readability were developed for English-language texts. Although each LLM was instructed to generate Chinese- and English-language texts independently, readability was assessed only for English-language texts. Therefore, the resulting English readability scores cannot reflect the actual reading difficulty of the Chinese-language texts and limit direct comparability between the 2 language versions. Future studies should develop and validate readability formulas specifically designed for Chinese-language medical texts. Second, the assessment of content quality relied primarily on ratings by reproductive medicine experts. Although expert evaluation helped ensure medical accuracy, comprehensiveness, and professional rigor, it might not have fully captured the actual experiences and perspectives of patients as the end users. In addition, the evaluation process did not include automated concordance checks against authoritative clinical guidelines or analyses of hallucination rates. Future studies should incorporate user evaluations involving patients with infertility and combine them with automated objective metrics to more comprehensively assess the real-world value of LLM-generated health education materials. Third, this study evaluated only 5 publicly accessible, general-purpose LLMs and did not include medical-specific LLMs, retrieval-augmented generation systems, or domain-optimized models. Therefore, the generalizability of the findings to other models remains to be established. Fourth, this study was based on standardized single-turn prompts and did not simulate multiturn dialogue or personalized contexts. However, in real-world clinical settings, patients with infertility often differ substantially in their health literacy levels and emotional needs. The standardized interaction paradigm used in this study may therefore have overestimated or underestimated the performance of LLMs in complex clinical contexts. Finally, this study focused solely on the quality of static text and did not conduct longitudinal research to evaluate the effects of these LLMs on long-term health outcomes among patients with infertility.

### Conclusions

The 5 LLMs differed significantly in the readability and content quality of the reproductive health texts they generated, and a trade-off may exist between these 2 dimensions. Among all 5 models evaluated, Kimi 2.5 demonstrated the best overall performance. Although its generated texts were associated with relatively high reading difficulty, it may still represent a preferred model for supporting health education in patients with infertility. The texts generated by DeepSeek-R1 showed strong performance in terms of content accuracy and comprehensiveness, but their readability was comparatively limited. The texts generated by Gemini 3 and Qwen 3 were more suitable for patients with infertility who have lower levels of health literacy, but their content quality remained insufficient. The relatively poor performance of the texts generated by ChatGPT (GPT-5.2 Plus) may be attributable to limitations in Chinese-language corpora, inadequate alignment with clinical guidelines, and insufficient adaptation to the Chinese cultural context. Overall, this study provided an evidence base for the selection of LLMs for health education for patients with infertility. Future research should further address the current challenge of balancing readability and content quality in LLM-generated texts.

## Supplementary material

10.2196/97878 Multimedia Appendix 1 Example of a standardized question with corresponding responses generated by the 5 large language models and post hoc pairwise comparisons of readability among the generated texts.

10.2196/97878 Multimedia Appendix 2 List of health education questions for patients with infertility.

## Abbreviations

ART: assisted reproductive technology
FKGL: Flesch-Kincaid Grade Level
FRE: Flesch Reading Ease
GFI: Gunning Fog Index
LLM: large language model
RHL: reproductive health literacy
RM-ANOVA: repeated-measures analysis of variance
