# Supplementary material for: Evaluating the Performance of Five Leading Large Language Models in Generating Health Education Materials for Patients With Infertility: Comparative Analysis
Source: J Med Internet Res. 2026 Oct 27;28:e97878. doi: 10.2196/97878 (PMC13727392; doi:10.2196/97878)
Supplement: Supplementary file 1 [file jmir-v28-e97878-s001.docx]

Table S1 Example of a standardized question and corresponding LLM-generated responses

| Primary structure | Secondary structure | Question | LLM | Chinese response | English response |
| --- | --- | --- | --- | --- | --- |
| 1. Physiological Risks and Disease Related Factors | Apply | 1.1 My doctor said that I may have infertility. What does this mean? Does it mean that I am unable to conceive naturally? | Deepseek-R1 | 医生说你可能有不孕症，意思是尝试怀孕一年后（如果年龄超过35岁，则是半年后）还没有怀上。这不等于完全无法自然怀孕。很多人被诊断为不孕症后，通过治疗或调整生活方式，仍然能自然怀孕。医生会帮你找出原因并制定计划。 | If the doctor says you might have infertility, it means you haven’t gotten pregnant after trying for one year (or six months if you’re over 35). It does notmean you can never get pregnant naturally. Many people diagnosed with infertility still get pregnant naturally with treatment or lifestyle changes. The doctor will help find the cause and make a plan. |
|  |  |  | GPT-5.2 Plus | 不孕症的意思是：在没有避孕的情况下，尝试怀孕一年以上还没有成功。这不等于一定不能自然怀孕。有些人只是怀孕更慢，或者需要一点帮助。 | Infertility means trying to get pregnant for one year without success.It does not mean you can never get pregnant naturally.Some people just need more time or help. |
|  |  |  | Kimi 2.5 | 是的，这很正常！ 就像赶公交车，看着车要开走了但你还没跑到站台，当然会着急。这种焦虑来自：生物钟的紧迫感（身体提醒时间有限）  ；怕错过机会（像错过打折活动）；担心身体变化（怕难度增加）。但焦虑就像乌云，会遮住阳光。你可以：  告诉自己“很多人都在这个时间上车”；看医生了解真实情况（可能比想象的好）；深呼吸，像等下一班车一样耐心。 | "Infertility" is like a math problem you haven't solved yet, but it doesn't mean you can never solve it. The doctor means you and your partner have been trying to get pregnant for one year (or six months if the woman is over 35), but it hasn't happened. This does NOT mean you can never get pregnant naturally. Many people with infertility later get pregnant naturally, or with simple treatments. It just means you need a doctor's help to find the reason, like getting a teacher's help with homework. |
|  |  |  | Qwen 3 | “不孕症”不是说“一定怀不上”，而是指：夫妻规律同房（没避孕）超过1年，还没怀孕，医生会说“可能有不孕问题”。就像手机信号弱，不等于没信号——可能是暂时的小故障，比如排卵晚了、精子游得慢一点，或者压力太大影响身体。大多数情况都能通过调整生活方式、吃药或小治疗来改善，很多人后来都自然怀上了哦！ | “Infertility” doesn’t mean “you can never get pregnant.” It just means: a couple has been trying (without birth control) for over 1 year, but no pregnancy yet. Think of it like a phone with weak signal not broken, just needing a little help! Maybe the egg came a bit late, or sperm need more energy to swim. With rest, healthy habits, or simple medicine, many people go on to get pregnant naturally. |
|  |  |  | Gemini 3 | 不孕症并不意味着你“永远不能”怀孕。它的意思是，如果你和伴侣尝试了一年（或者35岁以上尝试了半年）都没有成功，医生就需要介入帮忙了。这就像是一台机器卡住了，需要检查是哪里出了问题，通过治疗，很多人最后都能拥有自己的宝宝。 | Infertility doesn't mean you can "never" have a baby. It just means that after trying for a year (or six months if you are over 35), things aren't happening naturally. Think of it like a puzzle piece that isn't fitting yet; a doctor helps find the right piece so you can get pregnant. |
| 2. Family and Social Consequences | Understand | 2.5 Whenever I think about getting older and still not having a child, I feel extremely anxious. Is this a normal emotional response among people experiencing infertility? | Deepseek-R1 | 是的，这种焦虑在不孕人群中很常见。年龄增长可能带来生育能力下降的担心，加上社会时钟压力，容易感到着急。但焦虑是正常的情绪反应，不代表你不好。和伴侣、医生或心理咨询师谈谈，制定合理计划，可以帮助缓解焦虑。 | Yes, this anxiety is common among people struggling with infertility. Worries about declining fertility with age, plus societal "timeline" pressure, can make you feel rushed. But anxiety is a normal emotion — it doesn’t mean you’re failing. Talking with your partner, doctor, or a counselor, and making a realistic plan can help ease worries. |
|  |  |  | GPT-5.2 Plus | 这是不孕人群中非常常见的情绪。备孕失败也容易让夫妻紧张，因为：压力大，容易自责或互怪，情绪没地方放。 | Yes, this is very common for people facing infertility.  Trying for a baby can also strain a marriage because:  · Stress is high  · Guilt and blame increase  · Emotions build up  · Guilt and blame increase  Emotions build up |
|  |  |  | Kimi 2.5 | “不孕症”就像是一道数学题你没解出来，但不代表你永远不会做这道题。医生的意思是：你和伴侣已经尝试怀孕一年（如果女性35岁以上是半年），但还没有成功。这不等同于绝对不能自然怀孕。很多有不孕症的人后来都自然怀孕了，或者通过简单的治疗就能怀上。这只是说明你们需要医生的帮助来找出原因，就像做数学题需要老师指点一样。 | Yes, this is totally normal! It's like running for a bus that's about to leave but you haven't reached the stop yet — of course you're anxious. This anxiety comes from:  Biological clock urgency (body reminding time is limited), Fear of missing chances (like missing a sale)  Worry about body changes (afraid it gets harder) But anxiety is like dark clouds blocking sunshine. You can tell yourself "Many people catch the bus at this time"  See a doctor to know the real situation (might be better than you think) Take deep breaths and be patient like waiting for the next bus |
|  |  |  | Qwen 3 | 焦虑很常见！ 年龄焦虑像担心作业没写完——但身体有自己的节奏，不是“失败”。备孕失败常让夫妻吵架，因为压力像大雨：两人没伞，容易互相怪“你挡着我了”。其实你们是队友，不是对手！握紧手说“我们一起撑伞”，比指责更暖。 | Anxiety is super common! Age worries are like fearing unfinished homework — but bodies have their own rhythm, not “failure”. Trying-to-conceive struggles often cause couple fights because stress is like heavy rain — if two people share one umbrella, they might blame “you’re blocking me!”. But you’re teammates, not opponents! Hold hands and say “let’s share the umbrella”, it’s warmer than blaming. |
|  |  |  | Gemini 3 | 是非常正常的反应。我们都有一个心里的闹钟，年纪大了，闹钟就会响，提醒我们要抓紧时间。对于想要孩子却很难怀上的人来说，这个闹钟的声音听起来更吓人。几乎每个遇到这种情况的人都会担心时间不够用，你不是一个人在战斗。 | It is a very normal reaction. We all have a clock in our minds. As we get older, the alarm rings to tell us to hurry up. For people who want a baby but struggle to conceive, that alarm sounds much scarier. Almost everyone in this situation worries about running out of time. You are not alone. |

Table S2 Post hoc pairwise comparisons of readability among texts generated by the five LLMs

| Comparison | FRE | | | | GFI | | | | FKRL | | | |
| --- | --- | --- | --- | --- | --- | --- | --- | --- | --- | --- | --- | --- |
|  | MD | SE | 95% CI | p | MD | SE | 95% CI | p | MD | SE | 95% CI | p |
| DS vs CG | -12.233 | 1.757 | -17.285，-7.180 | <.001 | 4.346 | 0.369 | 3.284，5.408 | <.001 | 3.591 | 0.282 | 2.780，4.402 | <.001 |
| DS vs KM | -1.092 | 1.635 | -5.795，3.611 | >.99 | 0.844 | 0.310 | -0.049，1.736 | 0.078 | 0.574 | 0.285 | -0.245，1.394 | 0.468 |
| DS vs Q | -23.289 | 1.678 | -28.116，-18.462 | <.001 | 4.695 | 0.329 | 3.748，5.642 | <.001 | 3.589 | 0.291 | 2.751，4.428 | <.001 |
| DS vs G | -24.929 | 1.560 | -29.416，-20.443 | <.001 | 4.142 | 0.324 | 3.210，5.074 | <.001 | 3.656 | 0.283 | 2.841，4.471 | <.001 |
| CG vs KM | 11.141 | 2.002 | 5.383，16.898 | <.001 | -3.502 | 0.355 | -4.523，-2.482 | <.001 | -3.016 | 0.293 | -3.860，-2.173 | <.001 |
| CG vs Q | -11.056 | 2.058 | -16.977，-5.136 | <.001 | 0.349 | 0.430 | -0.888，1.586 | >.99 | -0.001 | 0.326 | -0.940，0.937 | >.99 |
| CG vs G | -12.697 | 1.946 | -18.295，-7.098 | <.001 | -0.204 | 0.356 | -1.229，0.821 | >.99 | 0.065 | 0.313 | -0.834，0.964 | >.99 |
| KM vs Q | -22.197 | 1.736 | -27.190，-17.204 | <.001 | 3.851 | 0.375 | 2.773，4.930 | <.001 | 3.015 | 0.325 | 2.080，3.950 | <.001 |
| KM vs G | -23.837 | 1.591 | -28.413，-19.262 | <.001 | 3.298 | 0.296 | 2.447，4.150 | <.001 | 3.082 | 0.276 | 2.287，3.876 | <.001 |
| Q vs G | -1.640 | 1.412 | -5.703，2.422 | >.99 | -0.553 | 0.296 | -1.405，0.298 | 0.648 | 0.067 | 0.251 | -0.654，0.787 | >.99 |

[Abbreviations](https://support.jmir.org/hc/en-us/articles/37982552280987-Submitting-Your-Manuscript-to-JMIR-Publications-A-Guide-for-Authors#h_01JXNF8F7NS395YSJ9GY501JE4): DS: DeepSeek-R1; CG: GPT-5.2 Plus; KM: Kimi 2.5; Q: Qwen 3; G: Gemini 3
