# Supplementary material for: Evaluating the Performance of Five Leading Large Language Models in Generating Health Education Materials for Patients With Infertility: Comparative Analysis
Source: J Med Internet Res. 2026 Oct 27;28:e97878. doi: 10.2196/97878 (PMC13727392; doi:10.2196/97878)
Supplement: Supplementary file 2 [file jmir-v28-e97878-s002.docx]

List of Health Education Questions for Patients with Infertility

| Primary structure | Secondary structure | Question | Source of the Indicators |
| --- | --- | --- | --- |
| 1. Physiological Risks and Disease Related Factors | Access | 1.1 My doctor said that I may have infertility. What does this mean? Does it mean that I am unable to conceive naturally? | ASRM ^[1]^ |
|  |  | 1.2 I have recently been experiencing severe menstrual pain, and I have heard that it may be caused by endometriosis. What is endometriosis, and could it affect my ability to become pregnant? | ESHRE ^[2]^ |
|  |  | 1.3 My doctor told me that I have primary ovarian insufficiency (POI). What exactly does this mean, and what are its typical symptoms? | ESHRE ^[3]^ |
|  |  | 1.4 I have been diagnosed with polycystic ovary syndrome (PCOS), and both my menstrual cycles and ovulation are irregular. What causes this condition? | ACOG ^[4]^ |
|  |  | 1.5 My husband and I have been trying to conceive for one year without success. What are the common causes of infertility in women and men, respectively? | ASRM ^[1]^ |
|  | Understand | 1.6 Both endometriosis and adenomyosis can cause dysmenorrhea. Are they the same condition, and do they have the same impact on fertility? | ESHRE ^[2]^ |
|  |  | 1.7 Besides affecting my ability to become pregnant, what long-term health risks may polycystic ovary syndrome (PCOS) pose? | ACOG ^[4]^、RCOG ^[5]^ |
|  |  | 1.8 I have been diagnosed with primary ovarian insufficiency (POI), and my menstrual cycles have become very irregular. Is it still possible for me to conceive naturally? | ESHRE ^[3]^ |
|  |  | 1.9 I am trying to conceive at an advanced maternal age (35 years or older), and my ovarian function has begun to decline. Is the decline in the quality and quantity of my eggs clearly associated with increasing age? | CDC ^[6]^ |
|  | Appraise | 1.10 If I become pregnant with twins or higher-order multiples through in vitro fertilization (IVF), what are the potential risks for the mother and the babies? | SART ^[7]^ |
|  |  | 1.11 I have been diagnosed with polycystic ovary syndrome (PCOS) and would like to understand how significantly it may affect ovulation. How can the difficulty and potential risks of conceiving naturally in my situation be assessed? | ACOG ^[4]^ |
|  |  | 1.12 I have been trying to conceive for several years, but examinations of my uterus, fallopian tubes, ovaries, and other reproductive organs have all shown normal results. What other factors should be evaluated to determine the possible cause of my infertility? | ASRM ^[1]^ |
|  |  | 1.13 I have a history of diabetes, and my blood glucose levels have remained unstable. How should the potential impact of diabetes on pregnancy be assessed before I try to conceive? | CPAM ^[8]^ |
|  | Apply | 1.14 I have heard that endometriosis may affect fertility. Are there any ways to reduce the risk of developing this condition? | ESHRE ^[2]^ |
|  |  | 1.15 I have been diagnosed with polycystic ovary syndrome (PCOS), and both my menstrual cycles and ovulation are irregular. How can medications or lifestyle modifications help improve my egg quality? | ACOG ^[4]^ |
|  |  | 1.16 My doctor said that my history of recurrent miscarriages has adversely affected my intrauterine environment. What dietary, exercise, or daily routine adjustments can I make to improve my ovulatory function? | Frequently Asked Questions from Patients  ^[9]^ |
|  |  | 1.17 There is a family history of primary ovarian insufficiency (POI), and I am concerned about a possible hereditary risk. When should I begin regular reproductive health evaluations for early monitoring and prevention? | ESHRE ^[3]^ |
|  |  | 1.18 I am male, and my partner and I have been trying to conceive for several years without success. What examinations should I undergo to assess my fertility? | ASRM ^[1]^、 CDC ^[6]^ |
| 2. Family and Social Consequences | Access | 2.1 What is “fertility-related stress,” and how does it typically manifest in family and social interactions? | ASRM ^[10]^ |
|  |  | 2.2 How might the expectations of family members and friends regarding childbearing influence my reproductive decisions? | Frequently Asked Questions from Patients  ^[9]^ |
|  |  | 2.3 When I see other people sharing photos or posts about their children on RedNote or WeChat Moments, I feel anxious and even inferior. Is this a normal reaction? | ASRM ^[1]^ |
|  |  | 2.4 In the context of the traditional Chinese belief in “carrying on the family line,” how should I cope with these pressures as a woman experiencing infertility? | Frequently Asked Questions from Patients  ^[9]^ |
|  | Understand | 2.5 Whenever I think about getting older and still not having a child, I feel extremely anxious. Is this a normal emotional response among people experiencing infertility? | ASRM ^[10]^ |
|  |  | 2.6 Can unsuccessful attempts to conceive easily cause tension in a couple’s relationship? What are the reasons for this? | ESHRE ^[11]^  Frequently Asked Questions from Patients  ^[9]^ |
|  |  | 2.7 Whenever I attend family gatherings, I feel excluded because I do not have children. Where does this feeling come from, and how should I understand and cope with it? | Frequently Asked Questions from Patients  ^[9]^ |
|  |  | 2.8 Rather than feeling excited, I feel more stressed about becoming a mother. Is this a normal way to feel? | Frequently Asked Questions from Patients  ^[9]^ |
|  | Appraise | 2.9 Is my current reproductive decision driven more by my personal wishes or by external expectations? How can I objectively assess the relative influence of these two factors? | Frequently Asked Questions from Patients  ^[9]^ |
|  |  | 2.10 Does my fertility-related anxiety mainly stem from family pressure, social comparison, or my own insecurities? | Frequently Asked Questions from Patients  ^[9]^ |
|  |  | 2.11 Can constantly comparing myself with others make my fertility-related stress worse? | Frequently Asked Questions from Patients  ^[9]^ |
|  |  | 2.12 Can fertility-related stress cause me to question my self-worth or the direction of my future life? | ASRM ^[1]^ |
|  | Apply | 2.13 How should I communicate with my parents so that they understand that whether to have children and when to have them are decisions for my husband and me to make? | Frequently Asked Questions from Patients  ^[9]^ |
|  |  | 2.14 How can my husband and I cope with fertility-related stress together and avoid blaming each other? | ASRM ^[10]^ |
|  |  | 2.15 How can I protect my mental health when facing societal pressure to have children? | ASRM ^[10]^ |
|  |  | 2.16 As a man, how can I manage my own psychological stress when my partner and I have been unsuccessful in trying to conceive, and how can I better support my partner during this process? | ASRM ^[10]^ |
| 3. Medical Interventions and Health Improvement (Examinations, Treatment, and Assisted Reproductive Technologies) | Access | 3.1 My doctor suspects that I may have endometriosis. Besides an ultrasound examination, what other tests should I undergo? | ESHRE ^[2]^ |
|  |  | 3.2 What treatment options are available for endometriosis? Can surgery cure it completely? | ESHRE ^[2]^ |
|  |  | 3.3 How is polycystic ovary syndrome (PCOS) diagnosed, and what treatment options are available? | ACOG ^[4]^、RCOG ^[5]^ |
|  |  | 3.4 I am only 28 years old and have been diagnosed with primary ovarian insufficiency (POI). My doctor has recommended hormone replacement therapy. Are there any side effects associated with long-term use? | ESHRE ^[3]^ |
|  |  | 3.5 My endometrium has remained thin and has not responded well to estrogen supplementation. What other methods can help increase endometrial thickness? | CFAS ^[12]^ |
|  |  | 3.6 My husband and I are considering assisted reproductive treatment, but we are unsure about the difference between in vitro fertilization (IVF) and intracytoplasmic sperm injection (ICSI). Which option would be more suitable for us? | SART ^[7]^ |
|  |  | 3.7 I would like to learn more about fertility preservation. What does it involve, and which groups of people should consider it? | ESHRE ^[13]^ |
|  |  | 3.8 If I meet the medical indications for using donor eggs or donor sperm, what steps are involved in the complete treatment process? | SART ^[7]^ |
|  | Understand | 3.9 If I do not plan to become pregnant soon, do I still need treatment for polycystic ovary syndrome (PCOS)? | ASRM ^[14]^ |
|  |  | 3.10 In what situations are assisted reproductive technologies such as in vitro fertilization (IVF), intrauterine insemination (IUI), and egg donation appropriate, and what are their approximate success rates? | SART ^[7]^  ASRM ^[1]^ |
|  |  | 3.11 My doctor said that ovulation induction requires an individualized treatment approach. What factors do doctors consider when adjusting the treatment regimen? | ACOG ^[4]^ |
|  | Appraise | 3.12 Each medical consultation is very brief. Is it possible to have the same doctor follow me throughout the entire course of treatment? | ESHRE ^[11]^ |
|  |  | 3.13 Will my doctor adjust the treatment plan over time based on my financial situation, psychological well-being, and physical response to treatment? | ESHRE ^[11]^ |
|  |  | 3.14 My doctor has recommended in vitro fertilization with preimplantation genetic testing (PGT), often referred to as “third-generation IVF” Under what circumstances is this approach recommended? | SART ^[7]^ |
|  | Apply | 3.15 I experience severe dysmenorrhea due to endometriosis. Could taking pain relievers such as ibuprofen affect ovulation or my chances of becoming pregnant? | ESHRE ^[2]^ |
|  |  | 3.16 Besides medication, can surgery help relieve the symptoms of polycystic ovary syndrome (PCOS), and are its effects long-lasting? | ACOG ^[4]^ |
|  |  | 3.17 What foods should I eat more of, and what dietary or lifestyle habits should I avoid, to potentially improve the success rate of in vitro fertilization (IVF)? | ESHRE [11] |
|  |  | 3.18 Should I take folic acid, coenzyme Q10 (CoQ10), or dehydroepiandrosterone (DHEA) during ovulation induction treatment? How should I choose among these supplements? | ESHRE ^[15]^ |
|  |  | 3.19 My wife is undergoing infertility treatment. Can I participate with her throughout the entire examination and treatment process, and what should I do to support and cooperate with her? | ASRM ^[14]^ |
| 4. Resource and Financial Constraints (Costs and Health Insurance Coverage) | Access | 4.1 Does China’s current medical insurance system cover the costs of infertility examinations and treatment? Are assisted reproductive technologies such as intrauterine insemination (IUI) and in vitro fertilization (IVF) covered? | ASRM ^[1]^ |
|  |  | 4.2 If I travel to a hospital in another city for infertility treatment, what procedures should I complete in advance? Is the medical insurance reimbursement rate different from that for treatment received locally? | Frequently Asked Questions from Patients  ^[9]^ |
|  |  | 4.3 Approximately how much does one complete cycle of in vitro fertilization (IVF) cost? | Frequently Asked Questions from Patients  ^[9]^ |
|  |  | 4.4 Are there any official financial assistance programs for infertility treatment, such as charitable funds or hospital fee-reduction policies? Are ordinary patients eligible to apply? | Frequently Asked Questions from Patients  ^[9]^ |
|  |  | 4.5 Are there any commercial insurance plans that cover infertility treatment? What policy terms and conditions should I pay particular attention to when purchasing such insurance? | ASRM ^[1]^ |
|  | Understand | 4.6 Why do the costs of infertility treatment vary among hospitals? Is a higher treatment cost directly associated with a higher treatment success rate? | Frequently Asked Questions from Patients  ^[9]^ |
|  |  | 4.7 What unexpected situations, such as ovarian hyperstimulation syndrome (OHSS) or cycle cancellation, may occur during treatment and lead to additional costs? | SART ^[7]^ |
|  | Appraise | 4.8 When comparing two hospitals, one is more expensive but has a higher success rate, while the other is less expensive but has limited outcome data. How should I weigh these factors when making a decision? | ESHRE ^[11]^ |
|  |  | 4.9 If my first IVF cycle fails, will I need to pay the full cost of the entire IVF process again for a second attempt? | Frequently Asked Questions from Patients  ^[9]^ |
|  |  | 4.10 Taking time off work or changing my job responsibilities for infertility treatment may reduce my income and affect my family’s finances. How should I decide whether making these sacrifices for treatment is worthwhile? | ESHRE ^[11]^ |
|  | Apply | 4.11 How should I communicate with my doctor to prioritize examinations and medications covered by medical insurance and reduce my out-of-pocket expenses? | ASRM ^[1]^  ESHRE ^[11]^ |
|  |  | 4.12 To avoid affecting my work and income, can I ask my doctor to schedule injections and egg retrieval on weekends or after working hours? | ESHRE ^[11]^ |
|  |  | 4.13 My family has recommended inexpensive folk remedies, such as secret traditional Chinese medicine formulas. How can I use my doctor’s advice to persuade them to support evidence-based medical treatment? | Frequently Asked Questions from Patients  ^[9]^ |
| 5. External Triggers (Online Information, Family Members, and Friends) | Access | 5.1 Which online sources of information about infertility treatment, such as hospital reviews and patients’ real-life IVF experiences, are relatively reliable? Can information publish on platforms such as DXY and the official WeChat accounts of Peking Union Medical College Hospital be considered trustworthy? | ESHRE ^[2]^ |
|  |  | 5.2 A friend shared her experience of achieving pregnancy after one IVF cycle. How can I determine whether her experience is relevant or applicable to my own situation? | ESHRE ^[11]^ |
|  |  | 5.3 Are there any formal support resources for people experiencing infertility in China, such as hospital-organized patient support groups or officially verified online communities? | ASRM ^[10]^ |
|  |  | 5.4 When I see media reports claiming that new stem cell therapies can treat infertility, what sources or channels should I use to verify whether these treatments are scientifically credible and clinically available? | Frequently Asked Questions from Patients  ^[9]^ |
|  | Understand | 5.5 Why do some patients achieve successful outcomes while others do not despite receiving the same treatment regimen? What are the key factors that influence treatment success? | SART ^[7]^ |
|  |  | 5.6 Why do the “miracle doctors” or folk remedies recommended by family members and friends often conflict with medical advice? What are the underlying reasons for these differences? | Frequently Asked Questions from Patients  ^[9]^ |
|  |  | 5.7 Why do doctors always say that other people’s experiences cannot be directly applied to my situation? How much do individual differences in medical conditions affect treatment decisions and outcomes? | ESHRE ^[11]^ |
|  | Appraise | 5.8 When faced with different suggestions, such as changing doctors, switching treatment plans, or trying traditional Chinese medicine, how can I determine which option is most suitable for me? | ESHRE ^[11]^ |
|  |  | 5.9 Will joining an infertility patient support group help relieve my stress, or could it make me feel more anxious? How can I evaluate its potential benefits and drawbacks? | ASRM ^[10]^ |
|  |  | 5.10 Online fertility supplements often claim to “improve implantation rates.” Are these claims consistent with medical evidence, and are such products worth trying? | Frequently Asked Questions from Patients  ^[9]^ |
|  | Apply | 5.11 When I read treatment experiences shared by other patients online, how can I identify the key information and discuss it effectively with my doctor? | ESHRE ^[11]^ |
|  |  | 5.12 When family members or friends insist on recommending treatments that are not appropriate for my condition, how can I clearly express my position while maintaining a good relationship with them? | Frequently Asked Questions from Patients  ^[9]^ |
|  |  | 5.13 To reduce misinformation and unnecessary distractions, which official accounts should I follow for reliable information about infertility, and how can I filter or block promotional content? | Frequently Asked Questions from Patients  ^[9]^ |
|  |  | 5.14 If participating in an infertility patient support group makes me feel more anxious, how should I adjust the way I engage with it, such as reading without posting or taking regular breaks from the group? | ESHRE ^[11]^ |
| 6.Coping Self-Efficacy and Psychological Adjustment | Access | 6.1 How can infertility and repeated IVF failures affect my mental health? | ASRM ^[1]^ |
|  |  | 6.2 I have been feeling persistently low and having trouble sleeping recently. Could this be due to excessive stress? | ASRM ^[10]^ |
|  |  | 6.3 Can negative emotions affect the success of ovarian stimulation, egg retrieval, or embryo transfer? | ASRM ^[10]^ |
|  |  | 6.4 As a man, how can I cope with the psychological stress associated with infertility? If I find it difficult to manage my emotions, how should I seek professional help? | ASRM ^[10]^ |
|  | Understand | 6.5 Will reproductive medicine doctors and nurses pay attention to my psychological well-being, or will they focus only on the medical and technical aspects of treatment? | ESHRE ^[11]^ |
|  |  | 6.6 If I experience significant psychological stress during infertility treatment, can the hospital provide psychological counseling or support? | ASRM ^[10]^ |
|  |  | 6.7 Are there any psychological support resources specifically designed for people experiencing infertility, such as online support groups, helplines, or books? | ASRM ^[10]^ |
|  | Appraise | 6.8 How can I tell whether my current low mood is temporary or requires professional intervention? | ASRM ^[10]^ |
|  |  | 6.9 After another unsuccessful treatment, should I push myself to continue treatment, or take a break to care for my emotional well-being? | ESHRE ^[11]^ |
|  |  | 6.10 My husband does not seem to fully understand the distress I am experiencing. Is this a common situation, and how should I communicate with him about it? | ASRM ^[10]^ |
|  | Apply | 6.11 What evidence-based methods can help me reduce the stress associated with infertility? | ASRM ^[10]^ |
|  |  | 6.12 Under what circumstances should I seek help from a mental health professional who specializes in infertility, and how can I identify reliable psychological support resources? | ASRM ^[10]^ |
|  |  | 6.13 Are there any authoritative organizations that provide free or low-cost psychological support? | ASRM ^[10]^ |

ASRM, American Society for Reproductive Medicine; ESHRE, European Society of Human Reproduction and Embryology; ACOG, American College of Obstetricians and Gynecologists; RCOG, Royal College of Obstetricians and Gynaecologists; CDC, Division of Reproductive Health, Centers for Disease Control and Prevention; SART, Society for Assisted Reproductive Technology; CPAM, Perinatal and Nutritional Metabolism Branch of the China International Exchange and Promotive Association for Medical and Health Care; CFAS, Canadian Fertility and Andrology Society.

**Source of the Indicators**

1.	American Society for Reproductive Medicine. FAQ About Infertility. Available from: <https://www.reproductivefacts.org/browse-resources/frequently-asked-questions/faq-about-infertility/>.

2.	European Society of Human Reproduction and Embryology. Information for women with endometriosis. 2022; Available from: <https://www.eshre.eu/-/media/sitecore-files/Guidelines/Endometriosis/ESHRE-ENDOMETRIOSIS-patient-Guideline_21032022.pdf>.

3.	European Society of Human Reproduction and Embryology. Information for women with iatrogenic premature ovarian insufficiency. Available from: <https://www.eshre.eu/-/media/sitecore-files/Guidelines/POI/ESHRE-POI-GUIDELINE_Patient-version_iatrogenic-POI.pdf>.

4.	American College of Obstetricians and Gynecologists. Polycystic Ovary Syndrome (PCOS). Available from: <https://www.acog.org/womens-health/faqs/polycystic-ovary-syndrome-pcos>.

5.	Royal College of Obstetricians and Gynaecologists. Polycystic ovary syndrome (PCOS): what it means for your long-term health. Available from: <https://www.rcog.org.uk/for-the-public/browse-our-patient-information/polycystic-ovary-syndrome-pcos-what-it-means-for-your-long-term-health/>.

6.	Centers for Disease Control and Prevention. Infertility: Frequently Asked Questions. 2024; Available from: <https://www.cdc.gov/reproductive-health/infertility-faq/index.html>.

7.	Society for Assisted Reproductive Technology. SART FAQ About IVF. Available from: <https://www.reproductivefacts.org/browse-resources/frequently-asked-questions/sart-faq-about-ivf/>.

8.	Perinatal and Nutritional Metabolism Branch of the China International Exchange and Promotive Association for Medical and Health Care. Chinese Expert Consensus on Preconception, Pregnancy, and Postpartum Management of Patients With Polycystic Ovary Syndrome. Available from: <https://drugs.dxy.cn/pc/clinicalGuidelines/ptZraqEbhwzLX-TGbknhclw>.

9.	Sun F, Yuan L, Fu AP, al. e. Frequently Asked Questions from Patients. 2025;40(20):38-43. doi: 10.3870/j.issn.1001-4152.2025.20.038.

10.	American Society for Reproductive Medicine. FAQ About the Psychological Component of Infertility. Available from: <https://www.reproductivefacts.org/browse-resources/frequently-asked-questions/faq-about-the-psychological-component-of-infertility/>.

11.	European Society of Human Reproduction and Embryology. What psychosocial support should you get from your fertility clinic? 2016; Available from: <https://www.eshre.eu/-/media/sitecore-files/Guidelines/Psychology/ESHRE-PSYCHOLOGY-GUIDELINE_Patient-version_15022016.pdf>.

12.	Canadian Fertility and Andrology Society. Management of thin endometrium in assisted reproduction. 2019; Available from: <https://cfas.ca/_Library/clinical_practice_guidelines/2019-_Thin_Endometrium_Guideline_RBMO.pdf>.

13.	European Society of Human Reproduction and Embryology. Information on female fertility preservation. 2020; Available from: <https://www.eshre.eu/-/media/sitecore-files/Guidelines/Female-fertility-preservation/ESHRE-FP-patient-Guideline_v2020_1.pdf>.

14.	American Society for Reproductive Medicine. Polycystic Ovary Syndrome (PCOS). Available from: <https://www.reproductivefacts.org/news-and-publications/fact-sheets-and-infographics/polycystic-ovary-syndrome-pcos/>.

15.	European Society of Human Reproduction and Embryology. Information on ovarian stimulation. 2025; Available from: <https://www.eshre.eu/-/media/sitecore-files/Guidelines/COS/2025/OS-guideline-update-2025_patient-leaflet.pdf>.
